# Metagenomic analyses of gut microbiome composition and function with age in a wild bird; little change, except increased transposase gene abundance

**DOI:** 10.1093/ismeco/ycaf008

**Published:** 2025-01-23

**Authors:** Chuen Zhang Lee, Sarah F Worsley, Charli S Davies, Ece Silan, Terry Burke, Jan Komdeur, Falk Hildebrand, Hannah L Dugdale, David S Richardson

**Affiliations:** School of Biological Sciences, University of East Anglia, Norwich, Norfolk, NR47TJ, United Kingdom; School of Biological Sciences, University of East Anglia, Norwich, Norfolk, NR47TJ, United Kingdom; School of Biological Sciences, University of East Anglia, Norwich, Norfolk, NR47TJ, United Kingdom; Quadram Institute, Norwich Research Park, Norwich, Norfolk, NR47UQ, United Kingdom; Ecology and Evolutionary Biology, School of Biosciences, University of Sheffield, Sheffield, S102TN, United Kingdom; Groningen Institute for Evolutionary Life Sciences, University of Groningen, 9718 BG, Groningen, The Netherlands; Quadram Institute, Norwich Research Park, Norwich, Norfolk, NR47UQ, United Kingdom; Groningen Institute for Evolutionary Life Sciences, University of Groningen, 9718 BG, Groningen, The Netherlands; School of Biological Sciences, University of East Anglia, Norwich, Norfolk, NR47TJ, United Kingdom; Nature Seychelles, Roche Caiman, Mahé, 1310, Republic of Seychelles, Seychelles

**Keywords:** gut microbiome, age, senescence, metagenomics, transposase, *Acrocephalus sechellensis*

## Abstract

Studies on wild animals, mostly undertaken using 16S metabarcoding, have yielded ambiguous evidence regarding changes in the gut microbiome (GM) with age and senescence. Furthermore, variation in GM function has rarely been studied in such wild populations, despite GM metabolic characteristics potentially being associated with host senescent declines. Here, we used 7 years of repeated sampling of individuals and shotgun metagenomic sequencing to investigate taxonomic and functional changes in the GM of Seychelles warblers (*Acrocephalus sechellensis*) with age. Our results suggest that taxonomic GM species richness declines with age and in the terminal year, with this terminal decline occurring consistently across all ages. Taxonomic and functional GM composition also shifted with host age. However, the changes we identified occurred linearly with age (or even mainly during early years prior to the onset of senescence in this species) with little evidence of accelerated change in later life or during their terminal year. Therefore, the results suggest that changes in the GM with age are not linked to senescence. Interestingly, we found a significant increase in the abundance of a group of transposase genes with age, which may accumulate passively or due to increased transposition induced as a result of stressors that arise with age. These findings reveal taxonomic and functional GM changes with age, but not senescence, in a wild vertebrate and provide a blueprint for future wild functional GM studies linked to age and senescence.

## Introduction

Senescence—a decline in physiological function in later life—occurs in most organisms [1, 2]. However, its onset and rate often differ greatly among individuals within populations [1, 3]. One factor that may contribute to individual differences in senescence is variation in host-associated microbial communities. The intestinal tract of animals contains a diverse collection of microbes and their genomes (the gut microbiome; GM), which play an important role in host adaptation and fitness [4, 5]. The GM influences the regulation of essential processes, such as digestion, reproduction, and immune function [6, 7]. However, shifts in GM composition can be detrimental to the host; certain microbes may be pathogenic, while overall dysbiosis may impair host function [8, 9].

Studies in humans and laboratory animals have shown that GM composition generally changes rapidly in early life [10, 11] before stabilizing during adulthood [12]. This is often followed by greater GM instability in advanced age including a loss of diversity and changes to composition [13–15]. These late-life compositional shifts are generally characterized by a loss of commensal or probiotic bacteria and an increase in pathogenic microbes [16]. GM functional changes with age have also been identified. For example, healthy aging has been associated with microbes that enable increased biodegradation and metabolism of xenobiotics [16, 17], whereas unhealthy aging has been linked to increased production of detrimental microbial metabolites [16].

Studies have demonstrated links between the GM and senescence in humans and laboratory animals, however, their GM composition varies markedly from their counterparts living in natural environments because of the artificial environments they are exposed to [18, 19]. It remains unclear if these effects can be generalized to wild animals [18–20].

Recent studies on wild organisms have not reached a consensus on what characterizes the aging microbiome. Some have documented altered GM composition [21–23], increased GM diversity [22, 24],, and reduced GM stability [25] with increasing age. Other studies have indicated that GM characteristics remain relatively stable throughout adulthood [25–27]. However, these studies have been based on 16S ribosomal ribonucleic acid (rRNA) gene metabarcoding, which is limited in resolution [28–30]. Shotgun metagenomic sequencing enables higher taxonomic resolution (species or strain level), as well as informing on the functional potential of microbial communities based on gene content [31–33]. In humans and captive primates, metagenomics has revealed an increase in pathogenic microbial genes, and a decrease in beneficial genes, with age [17, 34, 35]. To our knowledge, no previous studies have investigated GM functional changes with age and senescence using shotgun metagenomics in a wild population.

Also, most GM studies on wild animals have relied on a cross-sectional sampling of differently aged individuals [36–38] and, therefore, may be confounded by the selective appearance/disappearance of individuals with particular GM characteristics. A lack of longitudinal samples also makes it difficult to infer changes in GM stability with age [39]. Understanding what drives this GM variation is important, as it may lead to a deeper comprehension of the evolution of senescence and life-history trade-offs [3], and enhance our ability to prolong healthy lifespans. As senescence occurs at different rates across individuals, a longitudinal approach is crucial for accurately evaluating age-associated effects [40]. Given this rate variation, and because declines are expected to be greatest at the end of life, GM changes may be more closely associated with proximity to death than chronological age. Including such information in analyses requires accurate estimates of the point of death that are not confounded by dispersal.

The long-term study of the Seychelles warbler population on Cousin Island provides a powerful natural system to study GM variation and host senescence [3]. Its isolated nature allows for the longitudinal sampling of uniquely marked, known-age individuals across their entire lifespan and the collection of accurate survival and reproductive success data [41, 42]. Previous studies using 16S metabarcoding have demonstrated that Seychelles warbler GM composition is linked to subsequent survival [43] but identified no overall patterns of GM senescence [26]. Additionally, host age was not associated with GM diversity, but a very marginal effect of host age on GM composition was reported [26].

Here, we use shotgun metagenomics to assess fine-scale changes in the GM with age and senescence in the Seychelles warbler. First, we determine how GM taxonomic diversity and composition change with host age, particularly in a bird’s terminal year when GM dysregulation is expected to be at its greatest. Then we test the hypothesis that GM functional characteristics (assessed via microbiome gene content) will change with age, senescence, and in the terminal year.

## Materials and methods

### Study system and sample collection

Seychelles warblers are insectivorous passerines endemic to the Seychelles archipelago. The population on Cousin Island (29 ha; 04° 20′ S, 55° 40′ E) has been extensively monitored since 1985 in the winter (January–March) and summer (June–October) breeding seasons [3, 44, 45]. Each season nearly all new birds (offspring) are caught, in the nest or as dependent fledglings in the natal territory [45]. As many adult birds as possible are re-caught each season using mist nets. Bird age is determined using either lay/fledgling date [45] for the majority of individuals, if birds are first caught without a fledging date being recorded, eye color is used to estimate age instead (see [45]).

The population on Cousin Island consists of ca. 320 individuals grouped into ca. 115 territories, defended year-round by a dominant breeding pair [46, 47]. Territory quality is calculated each season using arthropod counts, vegetation density, and territory size information [45, 48].

Nearly every bird in the population (> 96% since 1997 [49]) has been caught and marked with a unique combination of a British Trust for Ornithology (BTO) metal ring and three plastic color rings, which enables them to be monitored throughout their lives [3, 50]. Individuals almost never disperse between islands and the annual resighting probability is ~98% ± 1% [41, 42, 51]. If an individual is not seen for two consecutive seasons it is assumed to have died (an error rate of 0.04%) [41, 42]. Death dates for individuals were set as the final day of the season in which the bird was last seen. Benign climatic conditions and a lack of predators result in relatively long-lived individuals (median lifespan 5.5 years, max lifespan 19 years) [46, 52]. Previous studies have found that male and female Seychelles warblers are sexually mature at 1-year-old, and senescence (survival and reproductive) begins at ca. 6 years of age [3, 41, 46, 53]. The annual survival of adults does not differ between sexes, remaining ~80% up to 6 years of age and then decreasing [3, 54]. Thus, there were no differences in survival senescence between the sexes [3, 46, 53]. In addition, elderly females in their last year of life (terminal year) had reduced reproductive success [55].

Fecal samples were collected from caught birds and stored as described previously (see [26]). Between 2017 and 2023 all caught birds were placed in a disposable flat-bottom waxed paper bag containing a sterilized plastic weighing tray underneath a sterilized metal grate [56]. This allows the bird to stand on the grate and feces to fall into the sterile tray, minimizing contact with the bird’s surface. After ca 15 minutes (after defecation) the bird was removed. The sample was collected, using a sterile flocked swab, and placed into a microcentrifuge tube containing 1 ml of absolute ethanol. Samples were stored at 4°C in the field before being transferred to −80°C for long-term storage. Contamination (hand) controls were collected from fieldworkers each season. The time-of-day that samples were collected and the number of days for which samples were stored at 4°C, were recorded. A ca 25 μl blood sample was also taken via brachial venepuncture and stored in 1 ml of absolute ethanol at 4°C.

### Deoxyribonucleic acid extraction and sequencing

Blood samples were processed with a salt extraction method [42] or Qiagen DNeasy Blood and Tissue Kit and the resulting deoxyribonucleic acid (DNA) was used for molecular sexing [52, 57].

DNA from fecal samples was extracted using the Qiagen DNeasy PowerSoil Kit with a modified protocol (see [56]). Samples were lysed using both mechanical agitation and enzymic processes [56]. Individuals for which multiple longitudinal samples were available were prioritized for metagenomic sequencing to capture within-individual changes. In total, 155 fecal samples from 92 individuals across 7 years were sequenced, as well as three positive controls (two extractions from a ZymoBIOMICS Microbial Community Standard [D6300], and one extraction from a ZymoBIOMICS Fecal Reference with TruMatrix™ Technology [D6323]), and six hand controls. Library preparation was performed in two lanes per run using the LITE protocol [58] and sequencing undertaken in two runs of 2 × 150 bp NovaSeq X platform. The D6300 extraction control was sequenced on both runs to compare extraction and batch effects.

### Bioinformatics

Shotgun metagenomic sequence analysis was carried out using the MATAFILER pipeline (see [5] and supplementary materials). Briefly, MATAFILER removes host reads, assembles reads, predicts, and annotates genes, builds metagenome-assembled genomes (MAGs) and metagenomic species (MGSs), and taxonomically assigned MGSs. Due to the high individuality of the Seychelles warbler GM and the high sequencing coverage required to assign MGS, Metaphlan4 was also used to taxonomically classify reads (see supplementary materials for details).

### Gut microbiome analyses

A total of 162 samples were successfully processed bioinformatically (153 fecal samples, 4 controls). Positive controls were successfully recovered, and hand controls did not contribute to substantial contamination in samples (Fig. S1).

The 153 fecal samples (Fig. S2) included 71 from 40 females and 82 from 51 males. In total, 41 individuals had one sample, 41 had two, eight individuals had three, and one individual had four samples. Age at sampling ranged from 0.6–17.0 years (mean 5.7 ± 0.3 SE). Of these, 48 were from 22 individuals in their terminal year (the year in which they died); with ages in terminal year ranging from 1.4–17.0 years. From all these samples, 1025 unique metaphlan4 species-genome-bins assignments were used for the subsequent taxonomic analysis (mean 29.3 ± 2.0 SE per sample).

All statistical analysis was performed using R version 4.33 [59, 60]. variance inflation factor scores (*car* version 3.1.2) were used to test for collinearity between variables in all models; all had a score < 3 indicating no issues with collinearity [61].

### Taxonomic gut microbiome changes with age

#### Taxonomic gut microbiome alpha diversity

A rarefaction curve of Metaphlan4 species was constructed with *iNEXT* version 3.0.1 to determine the read depth required to recover 95% of theoretically present species (Fig. S3) [62]. Taxonomic classifications were rarefied to a depth of 5500 reads before alpha diversity analysis; two samples were removed due to insufficient read depth. Species richness and Shannon diversity metrics were calculated per sample using R packages *phyloseq* version 1.46.0 and *microbiome* 1.24.0 [63, 64]. Wilcoxon rank sum tests were used to examine whether different sequencing plates affected species diversity (Shannon index, *P* = .353) and species richness (Observed index, *P* = .124), both were not significantly different.

A linear mixed effect model with a Gaussian distribution (lmer), and a generalized linear mixed effect model with a negative binomial distribution (glmer.nb), were used to model changes in species diversity (Shannon index) and richness (observed taxa), respectively, using *lme4* version 1.1–35.5 [65]. Fixed effect variables included in models were: host age (years); terminal year (yes/no); sex (male/female); breeding season (winter/summer); sample year (as a factor: 2017–2023); territory quality; storage days at 4°C (days); time of day collected (minutes since sunrise at 6:00 a.m.). Bird ID was included as a random effect.

Storage at 4°C in the field ranged from 4 days to 104 days (mean 36.3 ± 1.8 SE). A quadratic age term, and an interaction between terminal year and host age, were tested to assess whether GM changes became more extreme with age or if GM changes in the terminal year differ depending on age. These terms were dropped if not significant to allow interpretation of the main effects. Age was measured in years, but all samples taken when birds were > 12 years of age were designated as 12 years because these samples were rare (n = 9, max age = 17 years). Previous analysis shows that body condition is not associated with Seychelles warbler GM diversity and composition, thus, it was not included in analysis [43]. Model diagnostics were run using *DHARMa* version 0.4.6, with no significant issues in each chosen model [66]. Herein, all models were tested with the same variables unless stated otherwise.

A within-subject centering approach was used to separate between-individual (cross-sectional) GM differences with age (which could be driven by the selective appearance/disappearance of individuals with particular GM characteristics), from within-individual (longitudinal) change (which could indicate senescence) [67]. This involves calculating the mean age of each individual across all its sampling events (mean age) and the within-individual deviation from that mean age at each separate sampling event (delta age). These terms replace host age in the model. The fixed effect of terminal year was also replaced by a “terminal year bird” term (yes/no) which indicates whether individuals have at least one sample collected in the terminal year or not. An interaction between the terminal year bird and delta age, as well as quadratic delta age, were tested to assess whether within-individual GM changes were more extreme in birds with a sample taken in the terminal year of life and/or in older individuals, respectively (which would be indicative of senescence). In addition, an interaction between delta age and mean age was included in the models to test if within-individual changes with time occur differently depending on host age. The analysis was repeated with non-rarefied reads to determine if rarefaction influenced the results. These terms were dropped if not significant to allow interpretation of the main effects.

#### Taxonomic gut microbiome composition

A permutational multivariate analysis of variances (PERMANOVA) was carried out on a Euclidean distance matrix calculated using centered log ratio (CLR)-transformed reads, using the adonis2() function in *vegan* version 2.6.6 [68]. A blocking effect of Bird ID was used to account for repeated measures. The same predictors were included as for the main model in the Alpha diversity analysis above. Differences in composition were visualized with a principal component analysis (PCA) in *phyloseq* version 1.46.0 [64].

#### Taxonomic gut microbiome differential abundance analysis

Two different differential abundance analysis (DAA) methods were used to identify differentially abundant GM species with host age (as recommended by [69, 70]; *ANCOMBC2* version 2.4.0 and *GLLVM* version 1.4.3 [71, 72]. ANCOMBC2 calculates log fold change of species one at a time before adjusting p-values, whereas GLLVM calculates log fold change of all species all at the same time, accounting for correlation between species [71, 72]. A total of 22 common species, defined as species found in 20% of the population at >0.01% abundance, were retained. Species that were significantly differentially abundant in the same direction using both DAA methods were considered robustly significant. Variables included in each model were the same as in models above.

### Functional gut microbiome changes with age

#### Functional gut microbiome alpha diversity

Initially, 4727 different eggNOG orthologues (mean = 3616.6 ± 64.4 SE per sthe ample) were identified in our gene catalogs. A rarefaction curve of eggNOG orthologues was constructed using *iNEXT* to determine sample completeness [62]. Samples were then rarefied to 100 000 reads based on >95% completeness. One sample was removed due to insufficient reads. Following rarefication, 4685 eggNOG orthologues were retained (mean = 3054.3 ± 47.1 SE per sample). Due to the (negative) skewness of the observed richness and Shannon diversity of eggNOG annotations, a scaled exponential transformation and an exponential transformation were used for analyses, respectively, to improve residual fit. Both these alpha diversity indices were then analysed with linear mixed models containing the same predictors as for taxonomic alpha diversity above.

#### Functional gut microbiome composition

To test for changes in functional microbiome beta diversity, a PERMANOVA of Euclidean distances calculated from CLR-transformed read abundances per orthologue was used, using the same model structure as for taxonomic compositional analysis (described above). Differences in composition were visualized with a PCA plot as above.

#### Functional gut microbiome differential abundance analysis

DAA was performed on eggNOG annotations using their assigned categories from the database of clusters of orthologous genes (COG; Supplementary Table S1) [73] using *ANCOMBC2* and *GLLVM* as described above [71, 72]. Post-hoc DAA were performed on individual eggNOG members found within differentially abundant COG categories to establish the drivers of any significant differences (see Supplementary material for details).

## Results

### Taxonomic gut microbiome changes with age

#### Taxonomic gut microbiome alpha diversity

GM species richness declines with host age, and individuals in their terminal year had significantly lower species richness than those in a non-terminal year (Table S2 and Fig. S4). However, Shannon diversity was not significantly associated with host age, and did not differ between samples taken in a terminal or non-terminal year (Table S3). A quadratic age term, and an interaction between host age and terminal year were not significantly associated with species richness or Shannon diversity (*P* > .05) and were dropped from the final model.

**Figure 1:**
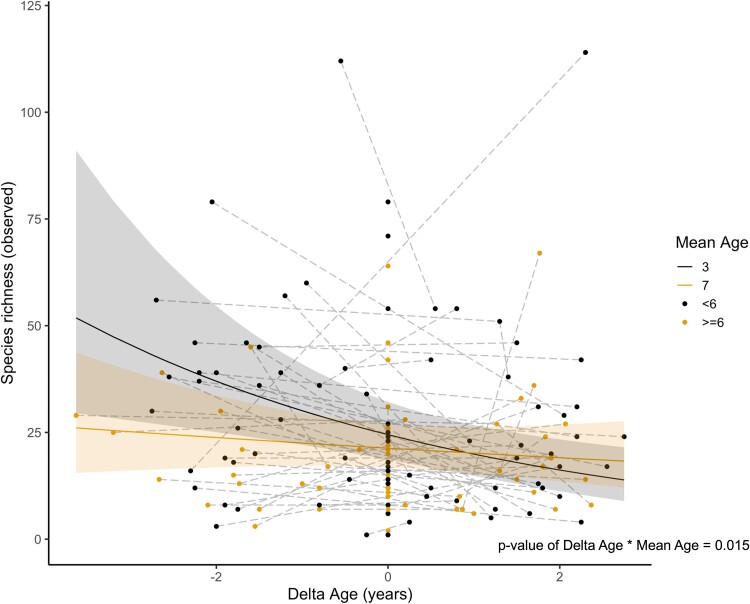
GM species richness in relation to within-individual, longitudinal differences in age (delta age in years) in Seychelles warblers. The solid lines represent model predictions with 95% confidence intervals calculated from the generalized linear mixed effect model (Table 1). Lines are model predictions at mean age of 3 and 7 before and after the start of senescence in this species [3]. Each point represents an individual GM sample, distinguished by mean age of <6 and greater or equal to 6, and the dashed lines connect samples from the same individual (*n* = 151 samples, 91 individuals).

The within-individual centering approach revealed that a decline in GM species richness with host age occurred longitudinally within individuals (Table 1, Fig. 1). However, the slope of declining species richness within an individual (delta age) decreases with increasing mean age, i.e. a decline in GM species richness with time occurs more at earlier host ages than in later life (Table 1, Fig. 1). Indeed, after the age of 6 there doesn’t appear to be any significant decline in GM species richness with increasing age (Fig. 1). This shows that contrary to our prediction that GM may show senescent effects, within-individual changes were less extreme in older individuals (in the ages we know senescence is occurring). There was also no evidence of between-individual selective disappearance effects (Table 1). Shannon diversity did not change significantly with mean or delta age (Table S4). There was also no evidence of a quadratic relationship between within-individual delta age and species richness or Shannon diversity, hence the quadratic age term was dropped from the final model. We also tested for an interaction between within-individual age and whether an individual’s final sample was in their terminal year, but this was not significant (*P* > .05) and was dropped. Additionally, the results were consistent with Table 1 when non-rarefied reads were used (Table S5). This result indicates that within-individual changes in species richness with age had a similar slope whether the bird was sampled in its terminal year or not.

**Table 1:** A generalized linear mixed effect model with a negative binomial distribution (glmer.nb) investigating GM species richness in relation to within-(delta) and between-(mean) individual variation in age among Seychelles warblers (*n* = 151 samples, 91 individuals). Conditional R^2^ = 53.1%. Reference categories for categorical variables are shown in brackets.

Predictor	Estimate	SE	*z*	*P*
**(Intercept)**	**2.705**	**0.317**	**8.536**	**<.001**
**Delta Age**	**−0.308**	**0.095**	**−3.233**	**.001**
Mean age	−0.036	0.023	−1.534	.125
Terminal year bird (yes)	−0.189	0.142	−1.329	.184
Season (winter)	0.020	0.157	0.126	.900
Sex (female)	−0.020	0.144	−0.139	.889
Days at 4°C	−0.238	0.137	−1.734	.083
Time of day	0.237	0.122	1.938	.053
Territory quality	−0.081	0.125	−0.645	.519
Sample year (2017)				
2018	0.439	0.280	1.568	.117
2019	0.399	0.323	1.233	.217
** 2020**	**0.701**	**0.351**	**1.997**	**.046**
** 2021**	**0.755**	**0.338**	**2.231**	**.026**
** 2022**	**0.725**	**0.346**	**2.099**	**.036**
** 2023**	**0.879**	**0.400**	**2.197**	**.028**
Delta age ^*^ mean age	**0.034**	**0.014**	**2.440**	**.015**
Random				
Individual ID	151 observations	91 individuals	Variance	.2321

*Note*: Significant (*P* < .05) predictors are shown in bold.

#### Taxonomic gut microbiome composition

A PERMANOVA analysis found that cross-sectional host age was a marginally significant predictor of GM taxonomic composition (Table 2), but terminal year was not (Table 2). Sample year, season, and catch time were significant and explain the largest proportion of GM compositional variance (Table 2) followed by days sample stored at 4°C and sex. An interaction between age and terminal year was not significant (*P* > 0.05). A PCA showed limited sample clustering according to age, which is consistent with the small amount of variance explained in the PERMANOVA (Fig. S5).

**Table 2:** A PERMANOVA analysis of GM taxonomic composition in relation to age and terminal year in the Seychelles warbler. The PERMANOVA was performed using a Euclidean distance matrix of CLR-transformed taxon abundances. *N* = 153 samples from 91 individuals. Bird ID was included as a blocking factor.

Predictor	*df*	R^2^	F	*P*
**Age**	**1**	**0.009**	**1.368**	**.043**
Terminal year	1	0.007	1.051	.569
**Season**	**1**	**0.013**	**2.021**	**.001**
**Sample year**	**6**	**0.056**	**1.479**	**<.001**
**Sex**	**1**	**0.007**	**1.096**	**.064**
**Days at 4°C**	**1**	**0.008**	**1.193**	**.034**
**Time of day**	**1**	**0.010**	**1.583**	**<.001**
Territory quality	1	0.005	0.813	.982

*Note*: Significant (*P* < .05) predictors are shown in bold.

#### Taxonomic gut microbiome differential abundance analysis

Five of the 22 common GM species found in the Seychelles warbler population (i.e. in >20% individuals) differed significantly in relative abundance with age in the GLLVM analysis (*Escherichia coli*, *Lactococcus lactis*, *Brucella pseudogrignonensis*, *Lactococcus garvieae*, *Microbacterium enclense*), but none were differentially abundant with age in the ANCOMBC2 analysis (Fig. S6A and B). Similarly, six species were differentially abundant in the terminal year in the GLLVM analysis (*L. garvieae*, *Pantoea anthophila*, *E. coli*, *Rothia* sp AR01, *M. enclense*, *B. pseudogrignonensis*), but none were differentially abundant with terminal year in the ANCOMBC2 analysis (Fig. S6C and D). Thus, there is no clear consensus of significant variation in the abundance of specific GM species with age or in the terminal year.

### Functional gut microbiome changes with age

#### Functional gut microbiome alpha diversity

Alpha diversity of eggNOG gene orthologues declined significantly with host age for both observed richness and Shannon diversity metrics (Table S6, Fig. S7). Alpha diversity of eggNOG orthologues did not differ between terminal year and non-terminal year samples (Table S6). Additionally, the interaction between host age (or quadratic age) and terminal year was not significant (*P* > .05).

**Figure 2:**
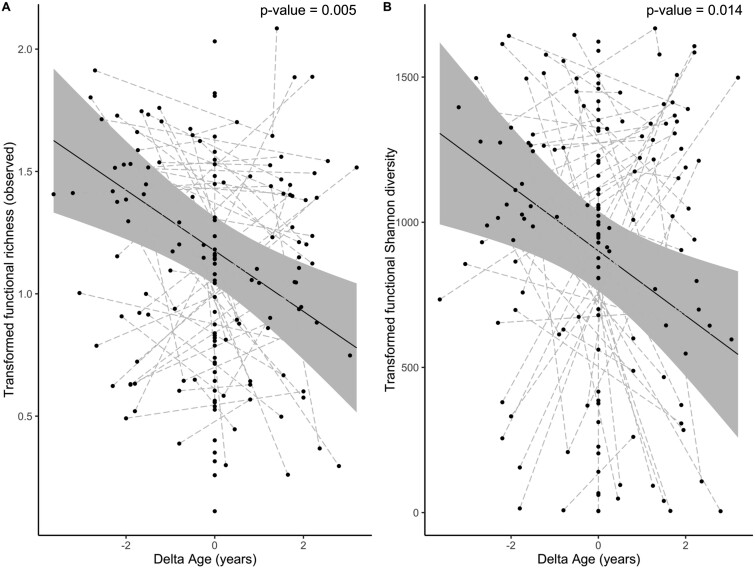
GM functional diversity measured as (A) observed richness and (B) Shannon diversity in relation to within-individual host age (years). Functional diversity calculations are based on eggNOG orthologue groups. Solid lines represent model predictions (± 95% confidence interval) from linear mixed effects models (Table 3). Each point represents a unique GM sample, and the dashed gray lines connect samples collected from the same individual (*n* = 152 samples, 90 individuals).

The decrease in functional alpha diversity with host age is best explained by within-individual longitudinal changes with age for both tested indices (Table 3, Fig. 2). Cross-sectional, between-individual age was a marginally significant predictor of Shannon diversity but not observed richness (Table 3). Alpha diversity did not differ between individuals that had at least one sample taken in their terminal year and those that did not. The interaction of terminal year bird and within-individual age, quadratic within-individual age, and the interaction between within-individual age and mean age were also not significant (*P* > .05) predictors of either index. Sample year was a significant variable of both eggNOG observed richness and Shannon diversity.

**Table 3:** A linear mixed effect model investigating variation in GM functional diversity (observed richness and Shannon diversity) in relation to within-(delta) and between-(mean) individual age in Seychelles warblers (*n* = 152 samples, 90 individuals). Functional diversity is based on eggNOG annotations. Observed richness and Shannon diversity were transformed using a scaled exponential and exponential function, respectively. Conditional R^2^ = 35.6% and 13.7%, respectively. Reference categories for categorical variables are shown in brackets.

**Observed richness**					
Predictor	Estimate	SE	*df*	*t*	*P*
**(Intercept)**	**0.99**	**0.17**	**124.77**	**5.68**	**<.001**
**Delta age**	**−0.12**	**0.04**	**137.00**	**−3.31**	**.001**
Mean age	−0.03	0.01	89.42	−1.97	.052
Terminal year bird (yes)	0.01	0.08	83.34	0.17	.870
Season (winter)	−0.06	0.10	136.94	−0.64	.525
Sex (female)	−0.06	0.08	81.33	−0.79	.430
**Days at 4°C**	**−0.19**	**0.09**	**127.35**	**−2.23**	**.028**
Time of day	−0.07	0.08	137.00	−0.88	.381
Territory quality	−0.07	0.08	129.62	−0.88	.381
Sample year (2017)					
2018	0.13	0.15	135.76	0.82	.416
2019	0.08	0.18	135.88	0.46	.647
2020	0.36	0.20	136.54	1.82	.071
** 2021**	**0.39**	**0.19**	**136.94**	**2.04**	**.044**
** 2022**	**0.56**	**0.19**	**128.48**	**2.90**	**.004**
** 2023**	**0.57**	**0.23**	**122.81**	**2.50**	**.014**
Random					
Individual ID	152 observations	90 individuals	Variance	0.050	
**Shannon diversity**					
**Predictor**	**Estimate**	**SE**	***df***	**t**	***P***
**(Intercept)**	**757.59**	**182.06**	**119.47**	**4.16**	**<.001**
**Delta age**	**−117.01**	**41.06**	**135.71**	**−2.85**	**.005**
**Mean age**	**−27.30**	**13.54**	**83.56**	**−2.02**	**.047**
Terminal year bird (yes)	17.93	79.75	76.74	0.23	.823
Season (winter)	173.07	104.67	127.74	1.65	.101
Sex (female)	−4.98	80.46	69.67	−0.06	.951
Days at 4°C	−48.55	95.70	133.26	−0.51	.613
Time of day	−21.18	81.57	132.14	−0.26	.796
Territory quality	−0.74	85.97	136.99	−0.01	.993
Sample year (2017)					
2018	88.02	168.08	136.67	0.52	.601
2019	32.22	200.48	136.71	0.16	.873
2020	169.50	210.62	131.73	0.81	.422
** 2021**	**464.12**	**206.85**	**136.39**	**2.24**	**.026**
** 2022**	**484.95**	**202.78**	**124.82**	**2.39**	**.018**
2023	453.37	238.55	116.14	1.90	.060
Random					
Individual ID	152 observations	90 individuals	Variance	5046	

*Note*: Significant (*P* < .05) predictors are shown in bold.

#### Functional gut microbiome beta diversity

A PERMANOVA analysis identified factors that were significantly related to GM functional composition (Table 4). Host age, but not terminal year, was a marginally significant predictor of functional composition (Table 4). An interaction between age and terminal year was not significant (*P* > .05). The largest effect sizes were found in relation to season, sample year, sex, and days stored at 4°C (Table 4). Time of day was not significant related to GM functional composition (in contrast to GM taxonomic composition). A PCA plot showed limited clustering of GM samples according to age, consistent with the small amount of variance explained by this variable (Fig. S8).

**Table 4:** A PERMANOVA analysis of GM functional composition in relation to age (and other factors) in the Seychelles warbler. The PERMANOVA was performed using a Euclidean distance matrix calculated using CLR-transformed (eggNOG) abundances. *N* = 153 samples; 91 individuals; bird ID was included as a blocking factor.

Predictor	df	R^2^	F	*P*
**Age**	**1**	**0.007**	**1.096**	**0.044**
Terminal year	1	0.006	0.890	0.292
**Season**	**1**	**0.011**	**1.823**	**0.042**
**Sample year**	**6**	**0.052**	**1.374**	**0.020**
**Sex**	**1**	**0.008**	**1.250**	**0.001**
**Days at 4°C**	**1**	**0.010**	**1.569**	**0.007**
Time of day	1	0.008	1.200	0.139
Territory quality	1	0.007	1.094	0.413

*Note*: Significant (*P* < .05) predictors are shown in bold.

#### Functional gut microbiome differential abundance analysis

**Figure 3:**
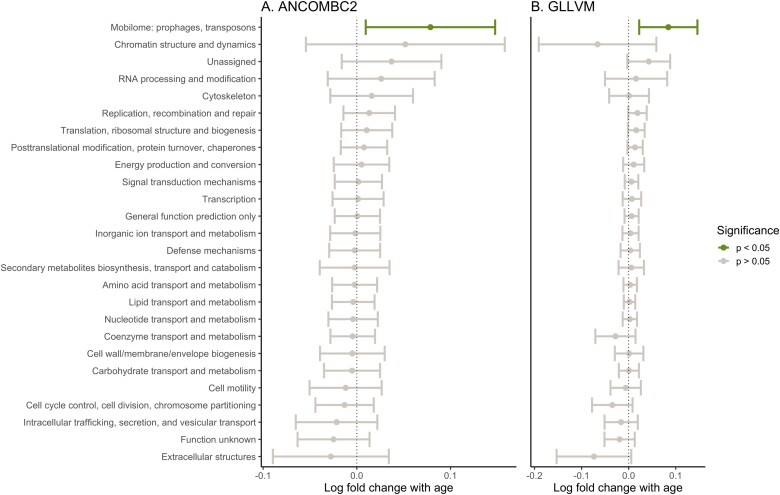
DAA of functional GM COG categories in Seychelles warblers using (A) ANCOMBC2 and (B) GLLVM. Each COG category is represented on the y-axis. Points and error bars are distinguished according to significance (*P* < .05).

Only one cluster of orthologous genes (COG) category was differentially abundant in relation to age. The COG category “X”, which represents mobilome COGs such as prophages and transposons, significantly increased in abundance with age in both the ANCOMBC2 and the GLLVM analyses (Fig. 3). Several COG categories were significantly differentially abundant with environmental variables including Cat A (RNA processing and modification) with season and Cat C (Energy production and conversion) with sample year (Figs S9 and S10).

**Figure 4:**
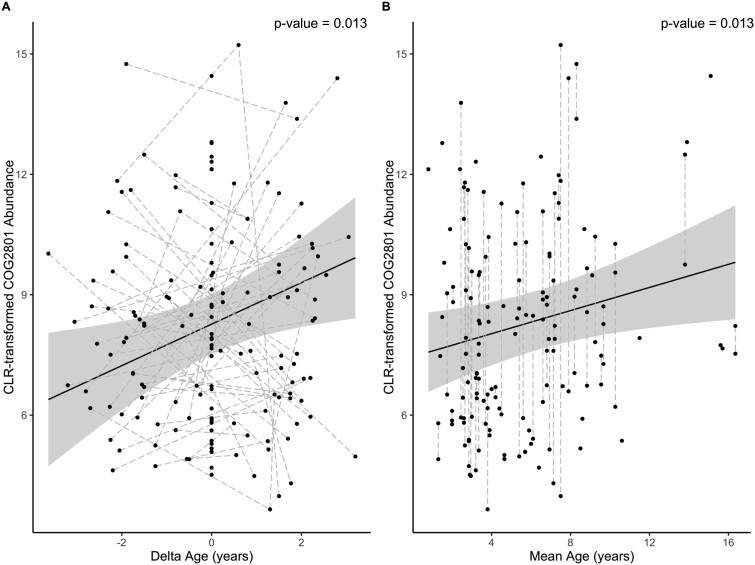
CLR-transformed COG2801 abundance in relation to (A) within-individual (delta) host age and (B) between-individual (mean) host age in the GM of Seychelles warblers. The solid line represents model predictions (± 95% confidence intervals) from a linear mixed effect model (Table S7). Each point represents a GM sample with dashed gray lines connecting samples from the same individual (*n* = 153 samples, 91 individuals).

Within category X (mobilome), only COG2801 (transposase genes) was found to significantly increase in abundance with age in both GLLVM and ANCOMBC2 analyses (Fig. S11, Table S1). A within-subject centering approach within a linear mixed model showed an increase in COG2801 was associated with both within-individual (longitudinal) age and between-individual (cross-sectional) age (Table S7, Fig. 4). However, the interaction between within-individual age and terminal year, as well as the interaction between within-individual age and mean age, was not significant (*P* > 0.05).

COG2801 located within MGSs (509 COG2801 copies from 160 MGS) were most closely related to the group insertion sequences (IS) 3 family of transposases (30%), other IS family transposases (12%), partial or putative transposases (33%) or other/unknown function (25%; Table S8). An increased abundance of COG2801 in the GM may be due to either an increase in the abundance of COG2801-carrying microbes or increased replication of the transposase gene itself. However, contrary to the first hypothesis, we found no relationship between the total abundance of COG2801-carrying MGSs (*n* = 160) and host age (Table S9). To further test this, COG2801-MGSs were matched with metaphlan4 annotations at the genus level; the abundance of COG2801-metaphlan4 genera was not significantly associated with host age (Table S10). Hence, the increase in COG2801 abundance with host age could not be attributed to an increased abundance of COG2801-carrying bacteria. Additionally, within COG2801, ten gene catalogs were commonly shared across 50% of samples. Each of these ten COG2801 gene catalogs was not significantly (p > 0.05) differentially abundant with age individually when tested using both ANCOMBC2 or GLLVM analysis (Fig. S12). Thus, the increase in abundance of COG2801 with age was not being driven by the abundance of a single prevalent, gene catalog but rather the cumulative abundance of many.

## Discussion

We used a repeated metagenomic dataset from individuals in a Seychelles warbler population to investigate how GM taxonomic and functional characteristics varied with host age. We identified a linear decrease in species richness, and small shifts in GM taxonomic composition, with host age. Additionally, species richness was lower in samples taken during an individual’s terminal year, but taxonomic composition did not differ between terminal and non-terminal samples. We also identified a linear decrease in the GM’s functional richness and diversity, and differences in functional GM composition, with host age. Finally, COG categories representing the mobilome increased in prevalence with bird age, driven by an increase in the abundance of COG2801, a group of transposases.

The small reduction in GM richness, but not Shannon diversity, with age suggests a loss of rare taxa that is not linked with a major restructuring of the evenness of the GM. The reduction in species richness was also age-dependent, with younger individuals experiencing greater reduction in species richness over time compared to older individuals, indicating that changes in GM species richness is not associated with senescence. This also concurs with the small changes in GM composition with age we identified; i.e. showing a limited number of differentially abundant taxa with increasing host age. This result is consistent with a previous 16S metabarcoding analysis of senescence of the Seychelles warbler GM despite the increased taxonomic resolution afforded by a metagenomics approach [26]. Additionally, the three dominant phyla identified in the metagenomics analysis (accounts for 95.6% of all taxonomic assignments) were the same three dominant phyla identified through the 16S analysis (Proteobacteria, Actinobacteria, and Firmicutes) [26, 43]. Overall, the results support the conclusion that, taxonomically, most of the GM stays the same with increasing age, apart from the loss of a few rare taxa.

Taxonomic changes in GM species diversity and composition with age have been repeatedly demonstrated in humans and captive animals [16]. However, in these species, late-life changes in the GM may be due to external factors such as antibiotic use, lifestyle, and dietary changes [18, 20]. An increasing number of wild animal studies are finding little evidence of a late-life shift in GM taxonomic diversity without such external factors (see [26,74]). Our study supports this conclusion despite the repeated sampling and increased resolution yielded by shotgun metagenomics, which can potentially reveal more nuanced changes at lower taxonomic levels.

Few studies have directly investigated functional changes in the GM with age in wild animals [75]. Some studies have been undertaken using functional inferences from metabarcoding sequence homology. However, this can be misleading due to being limited to variation within the same genus thus providing potentially inaccurate functional profiles. [76,77]. In our study using a higher resolution metagenomic approach, we found evidence of small, linear, changes in GM functional diversity and composition with age in the Seychelles warbler. Functional observed richness and Shannon diversity declined with age, which suggests not only that rare functions are lost, but that the evenness of these GM functions also changes linearly with adult age. Age-related decreases in functional richness and shifts in functional composition have previously been identified in elderly humans [78,79]. Such changes have been linked to the onset of specific disease states, such as inflammation and pathogenesis and changes to diet degradation and digestion, in humans and laboratory mice [80]. However, other studies have either found no change in functional alpha diversity, or even an increase in microbial functional richness and diversity with age [35,81]. Whether the loss of functional diversity, and minor changes in functional composition, with host age in Seychelles warbler is linked to declines in health and condition remains unclear and requires further study. The decline in taxonomic richness (but not taxonomic diversity) along with declines of functional richness and diversity with host age suggests that as the host age, less rare taxa contribute to the number and evenness of functional genes in the GM.

Despite the small changes in functional diversity and composition with age in the Seychelles warbler, we only identified one specific functional category whose abundance was significantly associated with host age. An increase in the abundance of COG2801 transposases occurred with age. However, this was not due to an increase in COG2801-carrying microbes. COG2801 are a group of transposases that are primarily found in bacteria (89.5%) and have been shown to be the most widely transferred genes among prokaryotes [82]. Most COG2801 genes found within MGSs were group IS3, which use a copy-out-paste-in mechanism to replicate [83]. This could lead to an increased number of transposon copies in the same individual bacterial genome over time, or to horizontally transfer to other bacterial genomes. [84,85]. Thus, the increased abundance of COG2801 with age in Seychelles warbler GM’s may be the result of self-replication, independent of microbial host cell DNA replication. An increase in transposition has been observed when bacteria are stressed and COG2801 is one of the most horizontally transferable eggNOG genes [86,87]. Therefore, as vertebrate hosts get older, the GM may be exposed to a greater number or intensity of stressors, such as mucus barrier thinning or inflammation, which may induce activation of COG2801 [88]. However, there was not an accelerated increase (i.e. a quadratic relationship) of COG2801 abundance with host age, which would be expected if the cumulative effects of host senescence were driving these changes. Therefore, stressors to the host that occur linearly in adulthood, such as cell death in the gastrointestinal autonomic nervous system [89,90], may better explain the increased abundance of COG2801 with host age.

We also focused on assessing terminal year effects in the Seychelles warbler GM. Only species richness was found to be significantly lower in the final year of a bird’s life. Moreover, the effect of terminal year was uniform across age, i.e. it was not more extreme in older individuals. Previous research has identified age-dependent terminal-declines in fitness components (reproductive success and survival probability) in the Seychelles warbler [55]. However, the lack of age-dependent terminal changes in GM characteristics identified in our study suggests that the GM does not undergo senescence in association with these other traits. As such, the declines in microbial species richness in terminal year samples (and linearly with age) may rather reflect the stabilization of the GM with age rather than a senescence effect. These results concur with the previous 16S metabarcoding analysis of the Seychelles warbler GM which found little evidence of GM senescence [26].

Across analyses, environmental factors explained most of the variance in the Seychelles warbler GM. This concurs with previous research on this species [26, 43, 56] as well as studies of other taxa [21,91,92]. Temporal variation -specifically year and season- explained the most variance in both taxonomic and functional GM composition. This may be explained by many factors including climate variability, differences in insect prey availability, or host population density [93–95]. Most Seychelles warbler individuals breed in the summer rather than the winter season, and GM shifts may therefore reflect reproductive activity and related hormonal changes [24]. Time of day was also associated with GM composition. Differences in insect activity might drive this pattern due to light availability and/or temperature [96,97]. However, such patterns could also be due to host intrinsic circadian rhythms [98]. In addition, differences in the amount of time samples were stored at 4°C resulted in differences in the GM characteristics and it is very important that these are controlled for. Given that samples are stored directly in absolute ethanol, the changes related to the time in storage at 4°C are likely to do with DNA degradation affecting the assignment of reads rather than an actual biological change in storage.

These factors lead to a substantial amount of noise in GM studies that can confound studies on aging, reproduction, and disease outcomes in wild populations. Therefore, accounting for these factors is important when investigating the GM in natural systems.

Our findings highlight the need for more studies investigating the functional characteristics of wild microbiomes as taxonomic relationships might not capture functional GM changes that occur (e.g. the increased prevalence of COG2801). However, researchers should not totally discount the utility of 16S metabarcoding for investigating general GM questions, as it may, in many cases, provide sufficient taxonomic resolution to answer specific questions [28]. Indeed, we identified similar taxonomic patterns using shotgun metagenomics to those revealed by a previous metabarcoding study on the Seychelles warbler [26]. The cost-effectiveness of 16S rRNA allows greater sample sizes, and thus power, to resolve certain questions. A combination approach that harmonizes both 16S metabarcoding and shotgun metagenomics has been proposed to maximize sample size, although such analyses are limited to genus-level comparisons [99]. On the other hand, shotgun metagenomics not only allows higher taxonomic resolution and functional analysis of the GM, but also an assessment of the interaction between taxa and their functions. As described with transposable elements, our functional analysis uncovered changes in GM function that were not detectable using 16S metabarcoding analysis.

In conclusion, while we found that the Seychelles warbler GM changes in terms of diversity, composition, and even function with age, this happens gradually over the adult lifespan and there is little evidence of late-life GM senescence. While species richness is lower in the terminal year, this occurs at all ages and is not more extreme in the oldest individuals. Interestingly, we found that the abundance of a group of transposase gene increases considerably with age in the GM, probably because of more frequent transposition within the GM community over time. Future work is required to determine exactly why these transposable element changes occur and what impact they may have. Additionally, work should investigate the generality of these conclusions by assessing whether functional changes occur in the GM of other wild vertebrates.

## Supplementary Material

Metagenomics_Senescence_Supplementary_Accepted_ycaf008

## Data Availability

All raw sequence data have been submitted to the European Nucleotide Archive database under the study accession numbers PRJEB81709.

## Code availability

The data files and script necessary to reproduce the statistical analysis and plots are provided at https://github.com/Chuen-Lee/SW_Senescence_GM
