# Supplementary material for: Metagenomic analyses of gut microbiome composition and function with age in a wild bird; little change, except increased transposase gene abundance
Source: ISME Commun. 2025 Jan 23;5(1):ycaf008. doi: 10.1093/ismeco/ycaf008 (PMC11833318; doi:10.1093/ismeco/ycaf008)
Supplement: Metagenomics_Senescence_Supplementary_Accepted_ycaf008 [file metagenomics_senescence_supplementary_accepted_ycaf008.docx]

**Supplementary Information:**

**Metagenomic analyses of gut microbiome composition and function with age in a wild bird; little change, except increased transposase gene abundance**

Wild gut microbiome changes with age

Chuen Zhang Lee^1*^, Sarah F. Worsley^1^, Charli S. Davies^1^, Ece Silan^2^, Terry Burke^3^, Jan Komdeur^4^, Falk Hildebrand^2^, Hannah L. Dugdale^4^, David S. Richardson^1,5*^

^1^ School of Biological Sciences, University of East Anglia, Norfolk, UK

^2^ Quadram Institute, Norwich Research Park, Norfolk, UK

^3^ Ecology and Evolutionary Biology, School of Biosciences, University of Sheffield, Sheffield, UK

^4^ Groningen Institute for Evolutionary Life Sciences (GELIFES), University of Groningen, Groningen, The Netherlands

^5^ Nature Seychelles, Roche Caiman, Mahé, Republic of Seychelles

*Correspondence: [david.richardson@uea.ac.uk](mailto:david.richardson@uea.ac.uk), chuen.lee@uea.ac.uk

David S. Richardson

School of Biological Sciences,

University of East Anglia,

Norwich Research Park,

Norwich,

NR47TJ,

United Kingdom

Chuen Zhang Lee

School of Biological Sciences,

University of East Anglia,

Norwich Research Park,

Norwich,

NR47TJ,

United Kingdom

**Supplementary methods**

Bioinformatics

Briefly, host reads were removed by mapping sequences to the Seychelles warbler genome (unpublished; complete BUSCO = 96.0% with a total length = 1,081,018,985 bp), using Kraken 2 (version 2.1.3). Remaining reads underwent quality filtering using sdm software version 2.14 beta [101,102]. After trimming, two samples and five hand controls were removed because they did not return enough reads for subsequent analysis (< 300,000 reads). An average of 20,481,040 (±1,109,059 SE) paired-end reads per sample were retained across the remaining samples.

The same trimmed reads were also used for *de novo* metagenome assembly, as implemented in MATAFILER: MEGAHIT version 1.2.9 [103] was used for metagenomic assemblies, on these genes were predicted using Prodigal version 2.6.3 [104] and clustered into a gene catalogue (95 % identity) of 19,527,109 gene clusters, and a gene abundance matrix created using rtk2 [105]. Functional annotations of clustered genes were done using eggNOGmapper version 2.1.12 and the evolutionary genealogy of genes: Non-supervised Orthologous Groups (eggNOG) database version 4 [82,106]. Subsequently, genome binning was done with SemiBin which created 4,176 bins (mean completeness = 34.95%, mean contamination = 1.41%) [107]. The bins were then filtered based on >80% completeness and <5% contamination using CheckM2 [108]; this retained 824 metagenome-assembled genomes (MAGs). MAGs were dereplicated across samples to generate 323 non-redundant metagenomic species (MGS) level bins, using clusterMAGs (https://github.com/hildebra/clusterMAGs). For MGSs, taxonomic assignment was performed using a marker-based approach with GTDB database version 214 [109]. Due to the high individuality of the warbler GM and the high sequencing coverage required to assign MGS, only one MGS was present in more than 50% of sequenced samples and relatively fewer MGSs were identified per sample (average 17 ± 1.3 SE per sample) which is likely to be an underestimate of the true diversity of the GM.

Therefore, Metaphlan4 version 4.1.0 (which is assembly-free and therefore requires lower coverage) was used to taxonomically classify reads using the default parameters [110]. Metaphlan4 assignments identified an average of 29.3 ± 2.0 species genome bins per sample and were used for the subsequent taxonomic analysis and MGS was only used for tracking functional annotations back to their taxonomy.

Post-hoc functional differential abundance analysis

Posthoc investigations were performed on individual eggNOG members found within the COG categories that were significantly differentially abundant with age. Firstly, a linear model was performed for each significant eggNOG member to test whether age-related changes were driven by between- or within-individual processes. Second, we tested if changes in the abundance of significant eggNOG members could be driven by changes in the abundance of the taxa from which these genes originate. To test this, the total abundance of MGSs carrying the eggNOG gene orthologs of interest was used as the response variable and age was included as a predictor in a lmer model. Furthermore, genera of eggNOG-carrying MGSs were matched with metaphlan4 genera to test whether the total abundance of known eggNOG-carrying genera was significantly associated with host age. Lastly, a protein-protein Basic Local Alignment Search Tool (BLASTp) analysis of each eggNOG gene ortholog of interest embedded within each MGS was performed to determine the identity of genes [111,112]. To test if the differential abundance of eggNOG members was driven by changes in the abundance of a specific gene (versus the cumulative abundance of many genes), gene catalogues assigned to the eggNOG cluster of interest (filtered to those with > 20% prevalence and 0.1% detection) were tested for differential abundance.

**Supplementary Figures and Tables**

Components of positive controls were successfully recovered as high-quality MGSs in acceptable relative abundances (Figure S2). Only 2 out of the 18 MGS from controls were found in faecal samples, both were widespread species *Enterococcus faecalis* and *Klebsiella pneumoniae* [113,114]. *E. faecalis* was part of the positive control but not found in the hand controls. *K.* *pneumoniae* was found in hand controls as well as samples but due to the low abundance in hand controls, we decided to retain all species for taxonomic analysis.


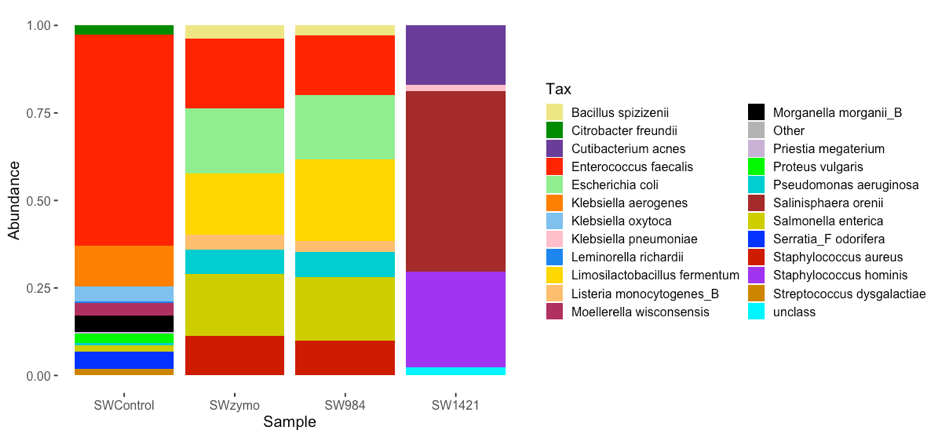


Figure S1. Controls and relative abundance of MGS at the species level. SWControl is positive control (ZymoBIOMICS Fecal Reference with TruMatrix™ Technology), SW984 and SWzymo are positive controls (ZymoBIOMICS Microbial Community Standard) sequenced separately, and SW1421 is a contamination (hand) control from 2023. We identified subspecies of *Bacillus subtilis* - *Bacillus spizizenii* and *Lactobacillus fermentum* – *Limosilactobacillus fermentum* . In SW1421 hand control, *Cutibacterium acnes* is linked to acne, *Klebsiella pneumoniae* is commonly found in the gut, *Salinisphaera orenii* are bacteria commonly isolated in high salinity environments, *Staphylococcus hominis* is commonly found to be harmless on human and animal skin.


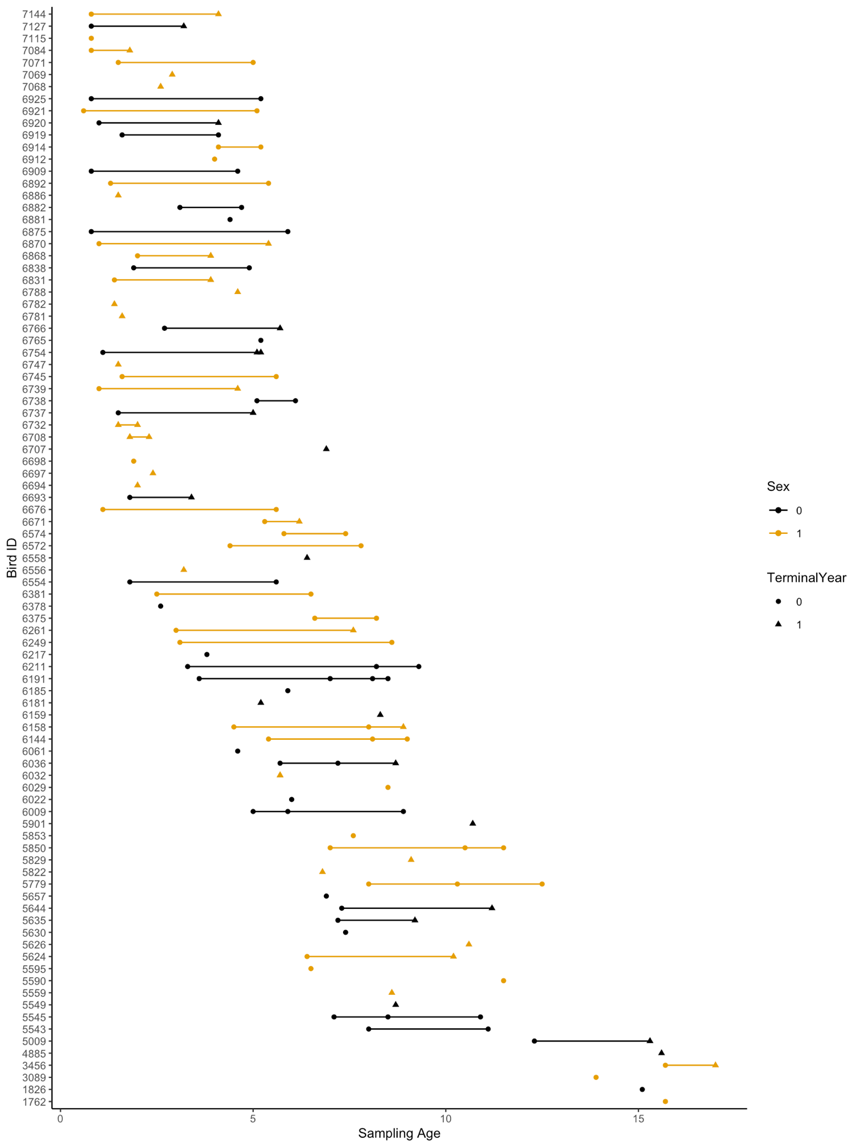


Figure S2. Seychelles warbler gut microbiome samples that were retained for analysis after sequencing and bioinformatics (n = 153 from 91 individuals). Points represent each sample, the x-axis represents individual’s age at sampling, whilst the y-axis represents individuals. Solid lines connect samples that were collected from the same individual. Colours represent the different sex (black = female, gold = male). Shape represents whether the sample was collected in the individual’s terminal year (circle = no, triangle = yes).

**Taxonomy**


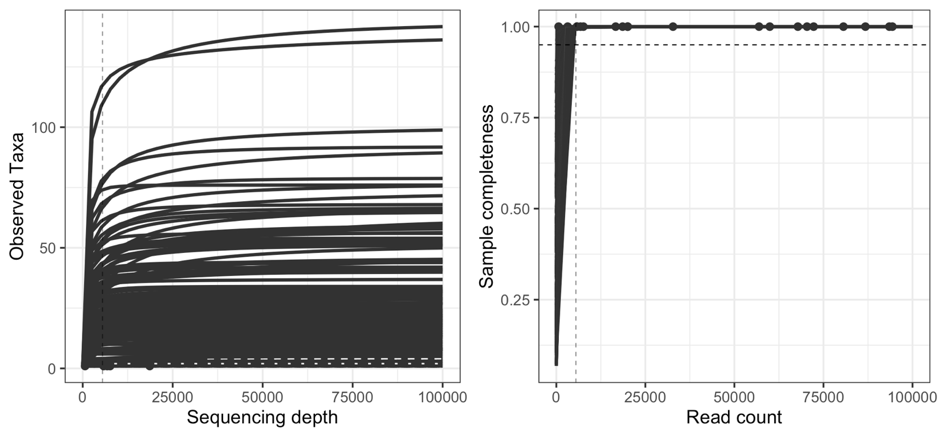


Figure S3. Sequencing depth against number of observed (metaphlan4) assembly-free taxonomic assignments (left) and read count against sample completeness (right) of each gut microbiome sample from Seychelles warblers (n = 153). 5500 reads at 95% completeness.

Table S1. COG functional categories [71]

| Abbreviation | COG Functional Categories |
| --- | --- |
| A | RNA processing and modification |
| K | Transcription |
| L | Replication, recombination and repair |
| B | Chromatin structure and dynamics |
| D | Cell cycle control, cell division, chromosome partitioning |
| V | Defense mechanisms |
| Y | Nuclear structure |
| T | Signal transduction mechanisms |
| M | Cell wall/membrane/envelope biogenesis |
| N | Cell motility |
| Z | Cytoskeleton |
| W | Extracellular structures |
| U | Intracellular trafficking, secretion, and vesicular transport |
| O | Posttranslational modification, protein turnover, chaperones |
| X | Mobilome: prophages, transposons |
| C | Energy production and conversion |
| G | Carbohydrate transport and metabolism |
| E | Amino acid transport and metabolism |
| F | Nucleotide transport and metabolism |
| H | Coenzyme transport and metabolism |
| I | Lipid transport and metabolism |
| P | Inorganic ion transport and metabolism |
| R | General function prediction only |
| Q | Secondary metabolites biosynthesis, transport and catabolism |
| S | Function unknown |
| ` | Unassigned |

Table S2. A generalised linear mixed effect model with a negative binomial distribution investigating the relationship between age, terminal year, and species richness in the gut microbiome of Seychelles warblers (n = 151 samples, 91 individuals). Significant (p < 0.05) predictors are shown in bold. Conditional R^2^ = 38.9%.

| Predictor | Estimate | SE | *z* | *P* |
| --- | --- | --- | --- | --- |
| (Intercept) | -125.20 | 71.62 | -1.75 | 0.081 |
| **Age** | **-0.04** | **0.02** | **-2.10** | **0.036** |
| **Terminal Year (yes)** | **-0.26** | **0.13** | **-2.06** | **0.039** |
| Season (winter) | 0.01 | 0.13 | 0.09 | 0.932 |
| Sex (female) | 0.01 | 0.13 | 0.05 | 0.959 |
| Time at 4℃ | -0.18 | 0.14 | -1.33 | 0.183 |
| Time of day | 0.22 | 0.12 | 1.82 | 0.069 |
| Territory quality | -0.08 | 0.12 | -0.67 | 0.506 |
| Sample Year | 0.06 | 0.04 | 1.79 | 0.073 |
| **Random** | | | | |
| Individual ID | 151 observations | 91 individuals | Variance | 0.14 |


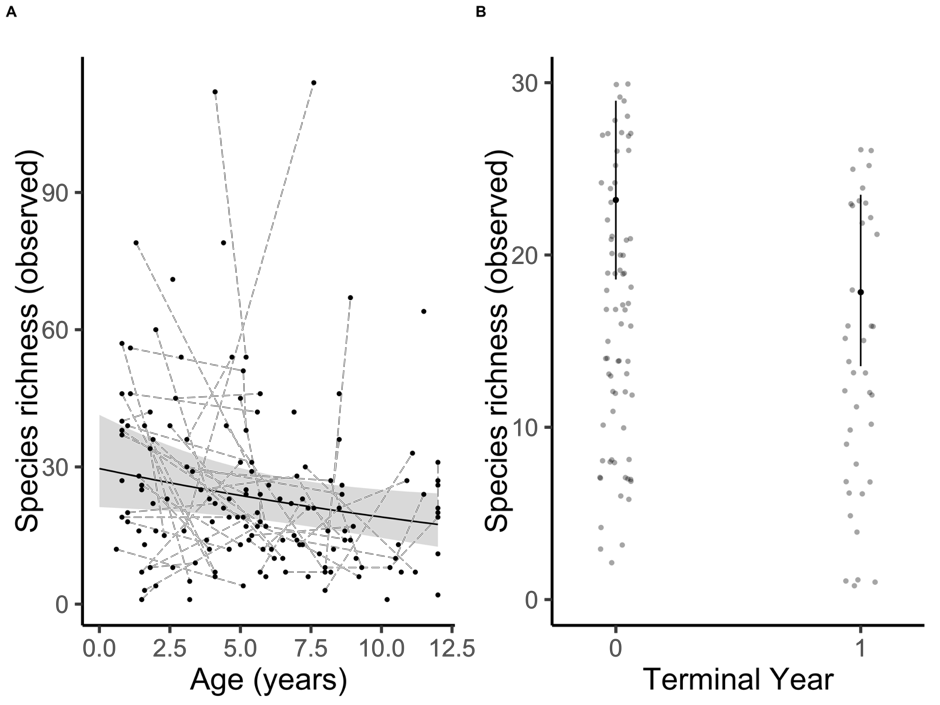


Figure S4. Species richness prediction from glmer.nb of the gut microbiome in the Seychelles warblers (n = 151 samples from 91 individuals). (A) Species richness against host age in years, solid black line and grey shaded area represent model predictions and confidence intervals respectively (Table S1, p = 0.036), points represent raw data. (B) Species richness against terminal year (0: No, 1: Yes), black dot and lines represent model predictions and error bars respectively, grey dots represent raw data points (Table S1, p = 0.039).

Table S3. A linear mixed effect model of Shannon diversity with chronological age and terminal year in the gut microbiome of Seychelles warblers (n = 151 samples, 91 individuals). Significant (p < 0.05) predictors are shown in bold. Conditional R^2^ = 46.4%.

| Predictor | Estimate | | SE | | *df* | | *t* | *P* |
| --- | --- | --- | --- | --- | --- | --- | --- | --- |
| **(Intercept)** | **-152.40** | | **76.85** | | **142.00** | | **-1.98** | **0.049** |
| Age | -0.01 | | 0.02 | | 86.36 | | -0.46 | 0.644 |
| Terminal Year (yes) | -0.16 | | 0.14 | | 133.79 | | -1.17 | 0.244 |
| Season (winter) | -0.12 | | 0.17 | | 130.60 | | -0.69 | 0.491 |
| Sex (female) | 0.10 | | 0.16 | | 74.64 | | 0.63 | 0.529 |
| **Time at 4℃** | **-0.32** | | **0.15** | | **113.36** | | **-2.15** | **0.034** |
| Time of day | -0.01 | | 0.13 | | 133.62 | | -0.10 | 0.920 |
| Territory quality | -0.14 | | 0.14 | | 124.33 | | -1.02 | 0.311 |
| Sample Year | 0.08 | | 0.04 | | 142.00 | | 2.00 | 0.047 |
| **Random** | | | | | | | | |
| Individual ID | | 151 observations | | 91 individuals | | Variance | | 0.27 |

Table S4. A linear mixed effect model of Shannon diversity within- and between- individual age analysis, accounting for subsequent close-to-death samples in the gut microbiome of Seychelles warblers (n = 151 samples, 91 individuals). Significant (p < 0.05) predictors are shown in bold. Conditional R^2^ = 49.7%.

| Predictor | Estimate | SE | *df* | *z* | *P* |
| --- | --- | --- | --- | --- | --- |
| **(Intercept)** | **0.95** | **0.35** | **129.65** | **2.75** | **0.007** |
| Delta Age | -0.07 | 0.07 | 135.41 | -1.12 | 0.265 |
| Mean Age | -0.18 | 0.16 | 77.16 | -1.14 | 0.257 |
| Terminal Year Bird (yes) | -0.01 | 0.03 | 81.30 | -0.24 | 0.809 |
| Sample Year | 0.09 | 0.06 | 105.90 | 1.60 | 0.11 |
| Season (winter) | -0.12 | 0.17 | 128.97 | -0.72 | 0.470 |
| Sex (female) | 0.10 | 0.16 | 75.58 | 0.62 | 0.535 |
| **Time at 4℃** | **-0.33** | **0.15** | **112.75** | **-2.24** | **0.027** |
| Time of day | -0.02 | 0.13 | 131.47 | -0.12 | 0.908 |
| Territory quality | -0.15 | 0.14 | 122.92 | -1.08 | 0.281 |
| Random | | | | | |
| Individual ID | 151 observations | 91 individuals | Variance | 0.3003 | |

Table S5. A linear mixed effect model of non-rarefied reads species richness within- and between- individual age analysis, accounting for subsequent close-to-death samples in the gut microbiome of Seychelles warblers (n = 151 samples, 91 individuals). Significant (p < 0.05) predictors are shown in bold. R2 = 0.4587057

| Predictor | Estimate | SE | *z* | *P* |
| --- | --- | --- | --- | --- |
| **(Intercept)** | **2.809** | **0.336** | **8.362** | **< 0.001** |
| **Delta Age** | **-0.320** | **0.105** | **-3.043** | **0.002** |
| Mean Age | -0.035 | 0.024 | -1.432 | 0.152 |
| Terminal Year  Bird (yes) | -0.170 | 0.147 | -1.161 | 0.246 |
| Season (winter) | 0.008 | 0.170 | 0.046 | 0.963 |
| Sex (female) | -0.063 | 0.149 | -0.424 | 0.672 |
| Days at 4℃ | -0.188 | 0.149 | -1.259 | 0.208 |
| Time of day | 0.240 | 0.132 | 1.819 | 0.069 |
| Territory quality | -0.099 | 0.136 | -0.724 | 0.469 |
| Sample Year (2017) | |  |  |  |
| 2018 | 0.461 | 0.297 | 1.552 | 0.121 |
| 2019 | 0.556 | 0.345 | 1.611 | 0.107 |
| **2020** | **0.851** | **0.373** | **2.283** | **0.022** |
| **2021** | **0.917** | **0.358** | **2.561** | **0.010** |
| **2022** | **0.834** | **0.362** | **2.302** | **0.021** |
| **2023** | **0.953** | **0.418** | **2.280** | **0.023** |
| **Delta Age * Mean Age** | **0.033** | **0.016** | **2.118** | **0.034** |
| Random | | | | |
| Individual ID | 153 observations | 91 individuals | Variance | 0.2119 |


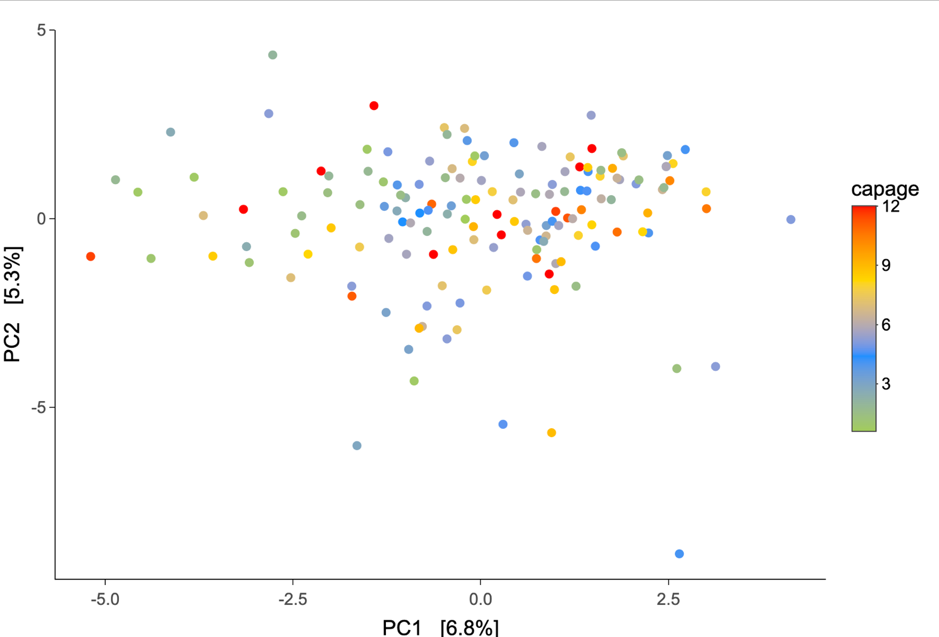


Figure S5. PCA plot of CLR-transformed reads in Euclidean distance, coloured by age.


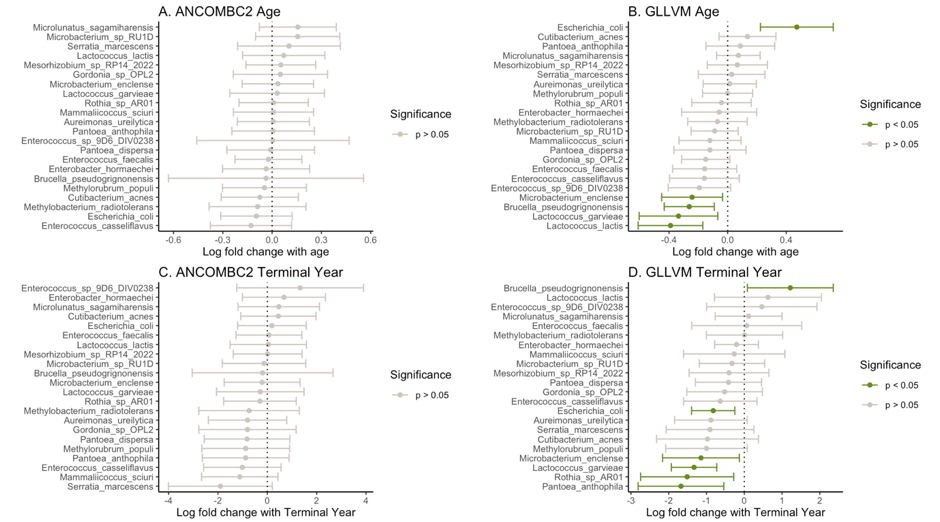


Figure S6. Taxonomic differential abundance analysis for common species (> 20% prevalence in the population). (A) ANCOMBC2 with age, (B) GLLVM with age, (C) ANCOMBC2 with terminal year, (D) GLLVM with terminal year. Significant (p < 0.05). Green = significant (p < 0.05) log fold change, grey = insignificant log fold change.

Table S6. A linear mixed effect model testing for age-related changes in functional scaled exponentially transformed observed richness and exponentially transformed Shannon diversity of eggNOG annotations in the gut microbiome of Seychelles warblers (n = 152 samples, 90 individuals). Conditional R^2^ = 33.7% and 9.2% respectively.

| **Observed Richness** | | | | | | | |
| --- | --- | --- | --- | --- | --- | --- | --- |
| Predictor | Estimate | SE | *df* | | | *t* | *P* |
| **(Intercept)** | -109.417 | 42.293 | 142.715 | | | -2.587 | 0.011 |
| **Age (years)** | **-0.036** | **0.013** | **92.620** | | | **-2.877** | **0.005** |
| Terminal Year (yes) | -0.124 | 0.077 | 142.784 | | | -1.605 | 0.111 |
| Season (winter) | -0.080 | 0.078 | 141.089 | | | -1.024 | 0.307 |
| Sex (female) | -0.080 | 0.080 | 78.890 | | | -1.008 | 0.317 |
| **Days at 4℃** | **-0.198** | **0.082** | **130.818** | | | **-2.422** | **0.017** |
| Time of day | -0.027 | 0.071 | 142.930 | | | -0.373 | 0.710 |
| Territory quality | -0.074 | 0.072 | 134.361 | | | -1.030 | 0.305 |
| **Sample Year** | **0.055** | **0.021** | **142.686** | | | **2.618** | **0.010** |
| Random | | | | | | | |
| Individual ID | 152 observations | 90 individuals | Variance | | | 0.047 | |
|  | | | | | | | |
| **Shannon Diversity** | | | | | | | |
| Predictor | Estimate | SE | *df* | | | *t* | *P* |
| (Intercept) | -92473.06 | 46119.45 | 143.00 | | | -2.01 | 0.047 |
| **Age (years)** | **-31.31** | **12.59** | **143.00** | | | **-2.49** | **0.014** |
| Terminal Year (yes) | -20.41 | 83.74 | 143.00 | | | -0.24 | 0.808 |
| Season (winter) | 105.32 | 85.76 | 143.00 | | | 1.23 | 0.221 |
| Sex (female) | -21.32 | 78.14 | 143.00 | | | -0.27 | 0.785 |
| Time at 4℃ | -36.85 | 92.11 | 143.00 | | | -0.40 | 0.690 |
| Time of day | 27.32 | 76.97 | 143.00 | | | 0.36 | 0.723 |
| Territory quality | -1.21 | 79.70 | 143.00 | | | -0.02 | 0.988 |
| Sample Year | 46.31 | 22.85 | 143.00 | | | 2.03 | 0.045 |
| Random | | | | | | | |
| Individual ID | 152 observations | 90 individuals | | Variance | 108.9 | | |


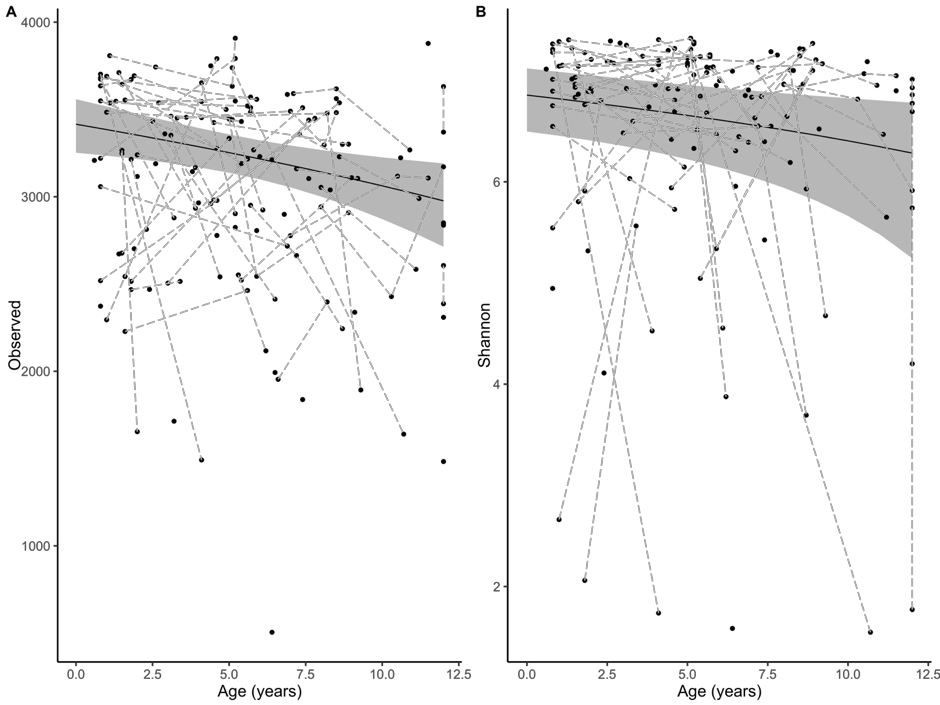


Figure S7. Evolutionary genealogy of genes: Non-supervised Orthologous Groups (eggNOG) (A) observed richness and (B) Shannon diversity against host age (years) model prediction from linear mixed effect model in the gut microbiome of Seychelles warblers (Table S4, p = 0.005 in A and p = 0.014 in B). The solid line represents model predictions and ribbon-shadding represent confidence intervals from model predictions. Each point represents a sample, and the dashed grey lines connect samples collected from the same individual (n = 152 samples from 90 individuals).


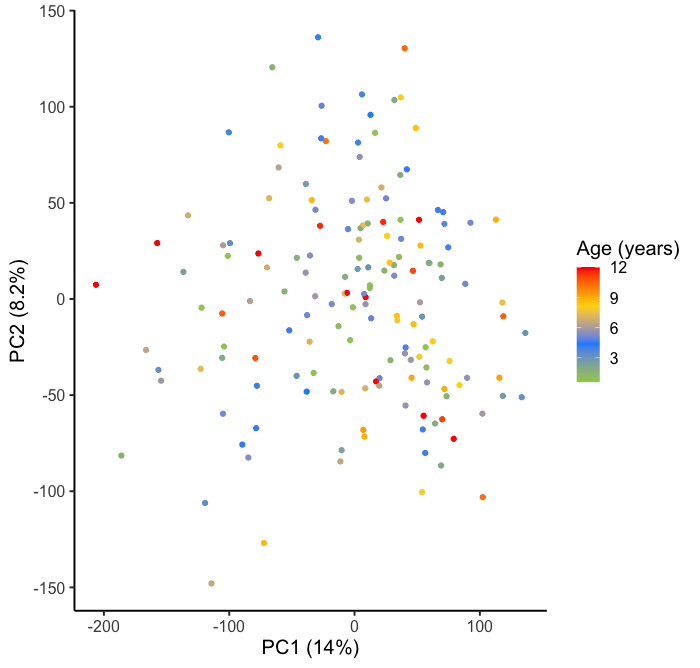


Figure S8. Functional PCA plot of CLR-count, euclidean distances of eggNOG annotations


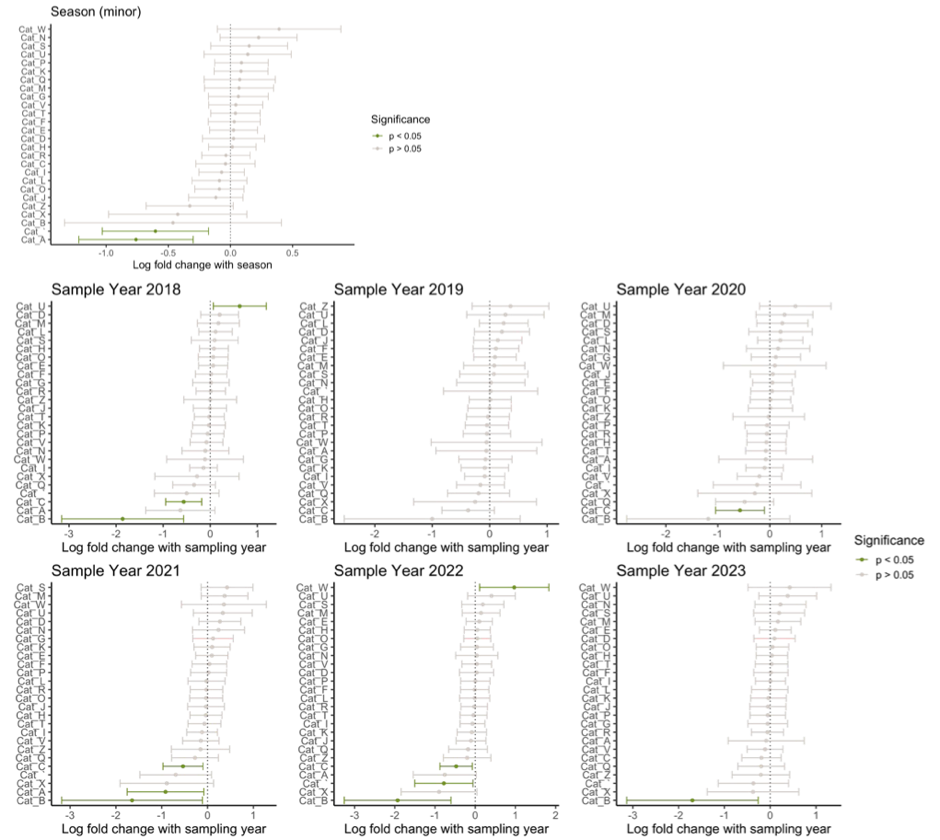


Figure S9. Differential abundance analysis of functional gut microbiome cluster of orthologous genes (COG) categories in Seychelles warblers using ANCOMBC2 with season and sample year. Each COG category is represented by a letter on the y-axis. Details of all COG categories are given in Table S5 [71]. “Cat_`” represents eggNOG annotations that were not assigned a COG category. Points and error bars are coloured according to significance (green: p < 0.05; grey: p > 0.05).


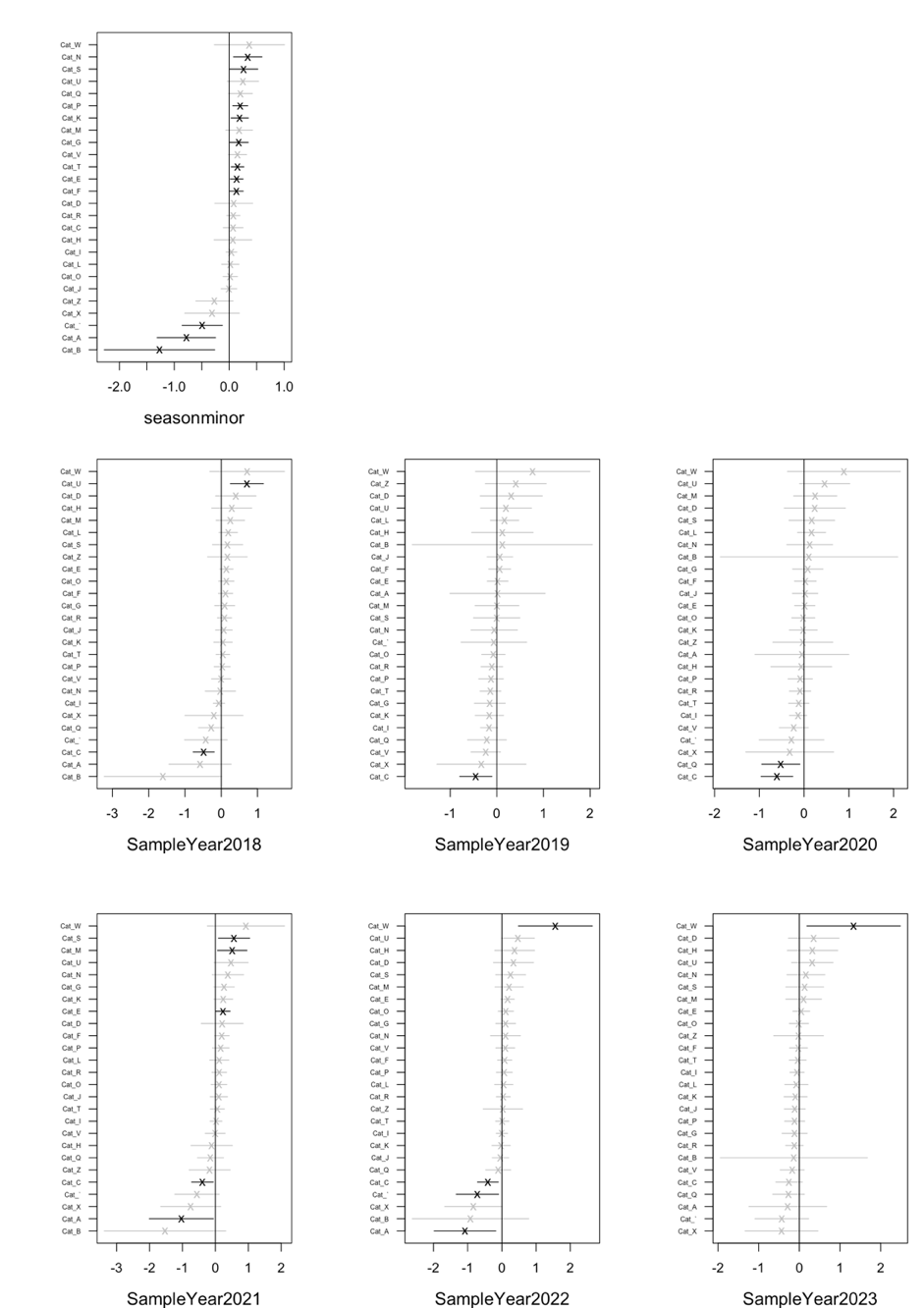


Figure S10. Differential abundance analysis of functional gut microbiome cluster of orthologous genes (COG) categories in Seychelles warblers using GLLVM with season and sample year. Each COG category is represented by a letter on the y-axis. Details of all COG categories are given in Table S5 [71]. “Cat_`” represents eggNOG annotations that were not assigned a COG category. Points and error bars are coloured according to significance (black: p < 0.05; grey: p > 0.05).


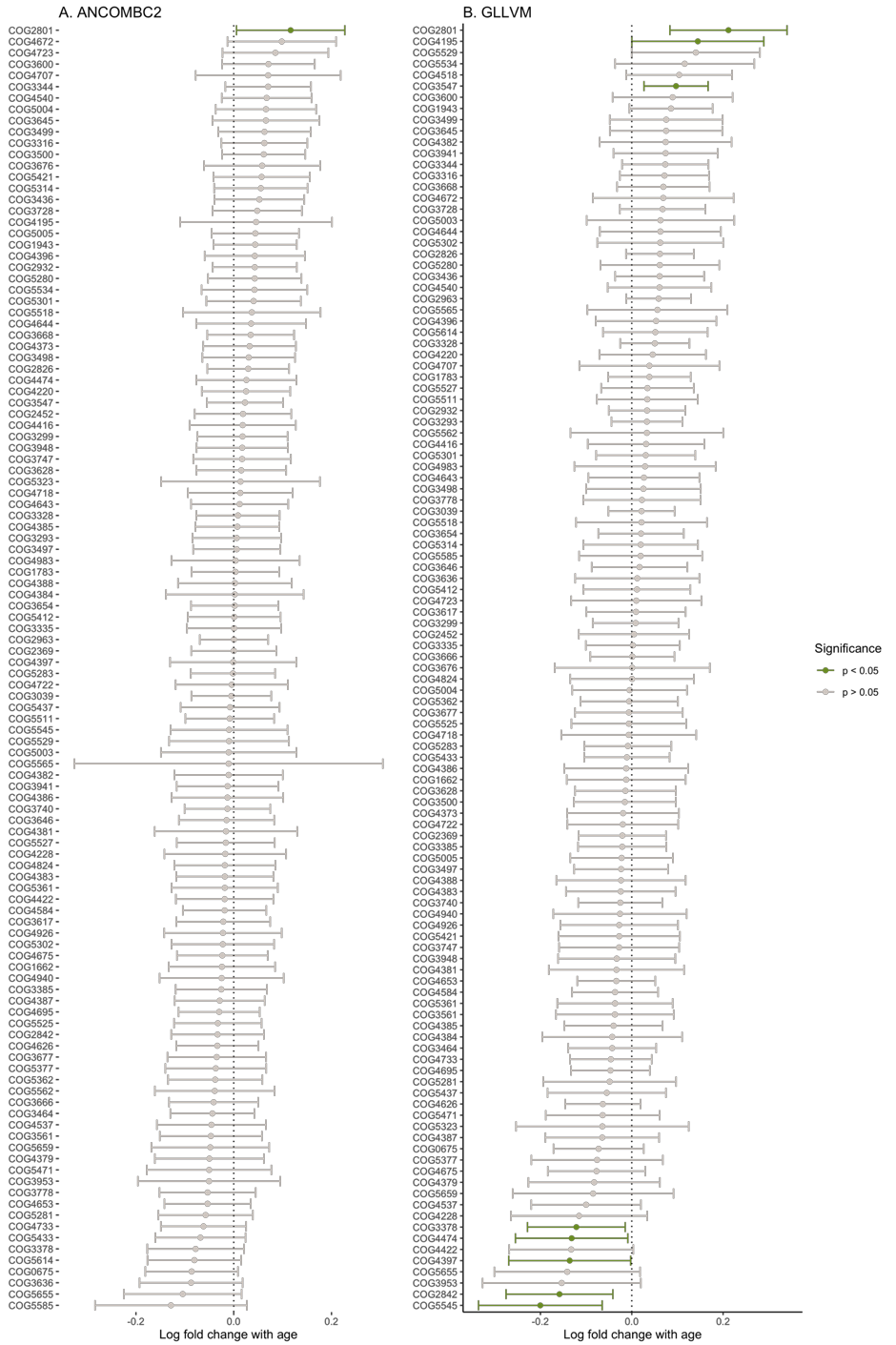


Figure S11 Differential abundance of COG X eggNOG members (A) ANCOMBC2 and (B) GLLVM.

Table S7. A linear mixed effect model of COG2801 abundance in the gut microbiome of Seychelles warblers in relation to within- (delta) and between- individual (mean) age. n = 153 samples, 91 individuals. Significant (p < 0.05) predictors in bold. Conditional R^2^ = 14.7%. Reference categories for categorical variables are shown in brackets

| Predictor | | Estimate | SE | df | t | *P* |
| --- | --- | --- | --- | --- | --- | --- |
| **(Intercept)** | | 9.700 | 0.971 | 115.370 | 9.989 | **< 0.001** |
| **Delta Age** | | **0.549** | **0.218** | **141.991** | **2.516** | **0.013** |
| **Mean Age** | | **0.157** | **0.062** | **85.606** | **2.534** | **0.013** |
| Terminal Year Bird (yes) | | 0.028 | 0.420 | 69.803 | 0.067 | 0.947 |
| Season (winter) | | -0.502 | 0.553 | 132.368 | -0.908 | 0.365 |
| Sex (female) | | 0.219 | 0.422 | 63.434 | 0.520 | 0.605 |
| Days at 4℃ | | -0.196 | 0.495 | 136.509 | -0.396 | 0.693 |
| Time of day | | -0.313 | 0.428 | 136.421 | -0.730 | 0.466 |
| Territory quality | | -0.315 | 0.452 | 141.901 | -0.697 | 0.487 |
| Sample Year (2017) | | |  |  |  |  |
| 2018 | | -1.662 | 0.902 | 140.921 | -1.844 | 0.067 |
| 2019 | | -1.457 | 1.068 | 141.645 | -1.363 | 0.175 |
| 2020 | | -2.200 | 1.129 | 134.384 | -1.949 | 0.053 |
| **2021** | | **-2.911** | **1.119** | **140.585** | **-2.601** | **0.010** |
| **2022** | | **-3.341** | **1.098** | **118.243** | **-3.042** | **0.003** |
| **2023** | | **-3.215** | **1.289** | **111.442** | **-2.495** | **0.014** |
|  | Random | | | | | |
| Individual ID | | 153 observations | 91 individuals |  | Variance | 0.1776 |

Table S8. BLASTp top hits for each COG2801 found in the genomes of all constructed metagenomics species (MGS) from the gut microbiome of Seychelles warblers (n = 153 from 91 individuals).

| **Top hit (contains keyword)** | **Count** | **Percentage** |
| --- | --- | --- |
| IS3 transposase | 154 | 30% |
| otherIS transposase | 64 | 13% |
| transposase | 170 | 33% |
| integrase | 30 | 6% |
| Mobile element protein | 4 | 1% |
| Helix-turn-helix | 19 | 4% |
| Hypothetical protein | 45 | 9% |
| Unknown | 23 | 5% |

Table S9. Linear mixed model on the CLR-transformed abundance of metagenomic species in the gut microbiome of Seychelles warblers (n = 2589 from 89 individuals). To test if COG2801-carrying MGS significantly differed in abundance with host age. Significant (p < 0.05) predictors are shown in bold. Conditional R^2^ = 46.9%.

| Predictor | Estimate | SE | *df* | t | *P* |
| --- | --- | --- | --- | --- | --- |
| (Intercept) | 5.44 | 0.41 | 233.52 | 13.34 | < 0.001 |
| Age | 0.03 | 0.04 | 69.32 | 0.79 | 0.432 |
| Terminal Year (yes) | 0.24 | 0.19 | 339.71 | 1.26 | 0.210 |
| Season (winter) | -0.09 | 0.22 | 394.36 | -0.43 | 0.671 |
| Sex (female) | 0.01 | 0.26 | 69.10 | 0.02 | 0.982 |
| **Time at 4℃** | **-0.44** | **0.18** | **434.74** | **-2.40** | **0.017** |
| **Time of day** | **-0.35** | **0.18** | **395.12** | **-2.01** | **0.045** |
| **Territory quality** | **-0.47** | **0.17** | **379.46** | **-2.85** | **0.005** |
| Sample Year (2017) |  |  |  |  |  |
| **2018** | **-0.77** | **0.41** | **402.12** | **-1.90** | **0.059** |
| **2019** | **-1.69** | **0.46** | **416.15** | **-3.71** | **0.000** |
| **2020** | **-1.19** | **0.48** | **360.43** | **-2.50** | **0.013** |
| 2021 | -0.70 | 0.46 | 334.48 | -1.53 | 0.127 |
| 2022 | -0.56 | 0.45 | 266.74 | -1.25 | 0.213 |
| 2023 | -0.65 | 0.49 | 239.09 | -1.33 | 0.186 |
| Random | | | | | |
| Individual ID | 874 observations | 85 individuals | | Variance | 1.042 |

Table S10. Linear mixed model on the CLR-transformed abundance of metaphlan4 genera in the gut microbiome of Seychelles warblers (n = 4477 from 91 individuals). To test if known COG2801-carrying genera significantly differed in abundance with host age. Significant (p < 0.05) predictors are shown in bold. Conditional R^2^ = 16.8%.

| Predictor | Estimate | SE | | df | t | *P* |
| --- | --- | --- | --- | --- | --- | --- |
| (Intercept) | 9.08 | 0.45 | | 316.13 | 20.37 | < 0.001 |
| Age | 0.04 | 0.04 | | 77.18 | 0.91 | 0.363 |
| Terminal Year (yes) | 0.30 | 0.22 | | 272.48 | 1.37 | 0.173 |
| Season (winter) | -0.30 | 0.27 | | 271.10 | -1.09 | 0.276 |
| Sex (female) | 0.15 | 0.27 | | 70.01 | 0.54 | 0.589 |
| **Time at 4℃** | **-0.52** | **0.22** | | **373.62** | **-2.34** | **0.020** |
| **Time of day** | **-0.60** | **0.21** | | **224.79** | **-2.82** | **0.005** |
| Territory quality | 0.03 | 0.21 | | 486.10 | 0.13 | 0.898 |
| Sample Year (2017) | | | | | | |
| 2018 | -0.15 | 0.47 | | 519.08 | -0.33 | 0.745 |
| 2019 | -0.85 | 0.54 | | 423.70 | -1.57 | 0.116 |
| 2020 | -0.80 | 0.55 | | 380.92 | -1.46 | 0.145 |
| **2021** | **-1.13** | **0.52** | | **377.58** | **-2.20** | **0.029** |
| 2022 | -0.56 | 0.49 | | 363.36 | -1.14 | 0.254 |
| 2023 | -0.06 | 0.55 | | 281.62 | -0.11 | 0.916 |
| Random | | | | | | |
| Individual ID | 1794 observations | | 89 individuals | | Variance | 0.995 |


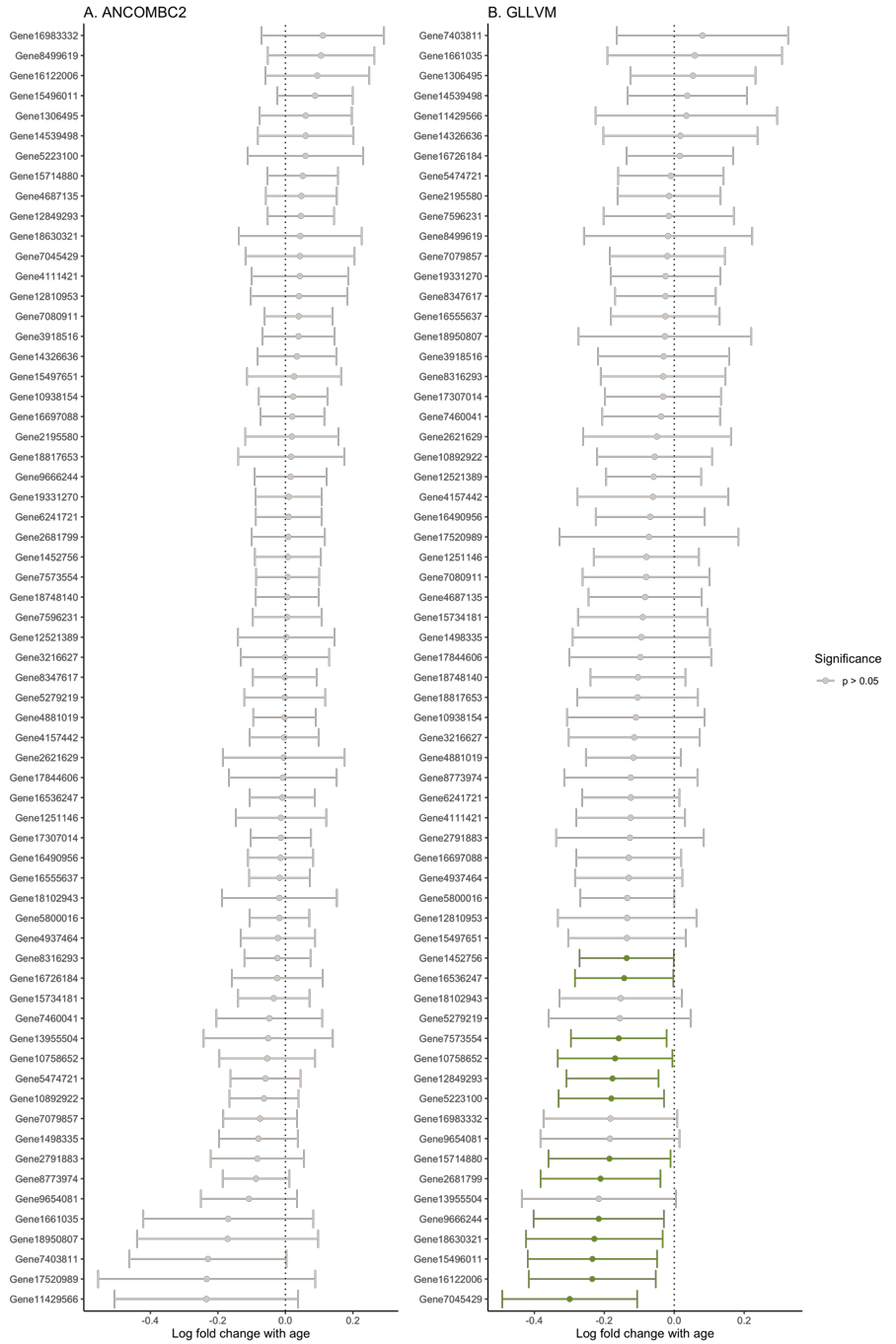


Figure S12. Differential abundance analysis of functional gut microbiome COG2801 gene catalogue that were commonly (20% prevalence) found in Seychelles warblers using (A) ANCOMBC2 and (B) GLLVM. Each gene catalogue (95% average nucleotide identity) are represented on the y-axis by their gene catalogue number. Points and error bars are coloured according to significance (black: p < 0.05; grey: p > 0.05).
